# Techniques of Inflow Occlusion for Liver Resection

**DOI:** 10.1155/1996/84618

**Published:** 1996

**Authors:** B. Launois

**Affiliations:** Centre Hospitalier & Universitaire de Rennes Rue Henri le Guilloux Rennes Cedex 35033 France

## Abstract

Limited resection can be a therapeutic approach in patients with cirrhosis with very low remnant hepatic function after resection. In this study, two hilar vascular clamping methods (hilar selective clamping [*n*=13] and hilar lobar clamping method [*n*=8]), which were used for resection ofhepatocellular carcinoma in patients with cirrhosis, were compared based on cardiovascular stability during clamping, intraoperative bleeding, operative time and postoperative course. In the past, the Pringle method had been used (*n*=19) and those instances were included for comparison. The mean operation time of the lobar clamping group was 209 ± 44 minutes, which was significantly less than that of the selective clamping group (259 ± 44 minutes, *p* < 0.05). Furthermore, the mean intraoperative blood loss of the lobar clamping group was 920 ± 400 milliliters, which was significantly less than that of the selective clamping group (1,640 ± 590 milliliters, *p* < 0.01). The postoperative total bilirubin and glutamine-oxaloacetic transaminase levels tended to be high in the Pringle group, but there was no significant difference between the groups. Although the blood pressure during clamping significantly decreased in all groups, the decrease was profound in the Pringle group as compared with those in the other two groups. Thus, as a method for controlling afferent bloodflow during hepatic resection in patients with cirrhosis, we recommend the lobar clamping method as a simple, safe and effective way to minimize bleeding and maintain cardiovascular stability.


### TECHNIQUES OF INFLOW OCCLUSION FOR LIVER RESECTION

### ABSTRACT

*Gotoh, M., Monden, M., Sakon, M., Kanai, T., Umeshita, K., Nagano, H. and Mori, T. (1994) Hilar lobar vascular occlusion for hepatic resection. Journal of the American College of Surgens; 178: 1–10.*

**Limited resection can be a therapeutic approach in patients with cirrhosis with very low remnant hepatic function after resection. In this study, two hilar vascular clamping methods (hilar selective clamping [$n$ = 13] and hilar lobar clamping method [$n$ = 8]), which were used for resection of hepatocellular carcinoma in patients with cirrhosis, were compared based on cardiovascular stability during clamping, intraoperative bleeding, operative time and postoperative course. In the past, the Pringle method had been used ($n$ = 19) and those instances were included for comparison. The mean operation time of the lobar clamping group was 209 ± 44 minutes, which was significantly less than that of the selective clamping group (259 ± 44 minutes, $p < 0.05$). Furthermore, the mean intraoperative blood loss of the lobar clamping group was 920 ± 400 milliliters, which was significantly less than that of the selective clamping group (1,640 ± 590 milliliters, $p < 0.01$). The postoperative total bilirubin and glutamine-oxaloacetic transaminase levels tended to be high in the Pringle group, but there was no significant difference between the groups. Although the blood pressure during clamping significantly decreased in all groups, the decrease was profound in the Pringle group as compared with those in the other two groups. Thus, as a method for controlling afferent bloodflow during hepatic resection in patients with cirrhosis, we recommend the lobar clamping method as a simple, safe and effective way to minimize bleeding and maintain cardiovascular stability. *J. Am. Coll.*, 1994, 178: 6–10.**

KEYWORDS: Liver resection technique hepatocellular carcinoma liver cirrhosis.

### PAPER DISCUSSION

The risk of severe intraoperative haemorrhage occuring when the liver parenchyma is divided has led to research into means of controlling the vascular inflow and outflow to the liver.

In 1908, J. Hogarth Pringle of Glasgow published a paper entitled "Notes on the arrest of hepatic haemorrhage due to trauma". This was the first time that total clamping of the hepatic pedicle in order to arrest haemorrhage had been proposed.

In hepatectomy, partial occlusion has long been practised by those who divided the hepatic pedicle structures outside the liver. However, Makuuchi has extended this technique to dissection of the hepatic pedicle structures outside the liver for clamping of the branches beyond the confluence without division. Thus in the removal of the right lateral sector of the liver, for example, the right hepatic artery and right branch of the portal vein are dissected and clamped. The principles of anterior intrahepatic approach were first elaborated by Ton That Tung. Here the liver parenchyma is divided along one of the fissures of the liver before the hepatic pedicles or veins are isolated. Most of the fissures of the liver have no external markings, and so the lines of dissection are usually estimates only of where the fissure lies. The hepatic pedicle structures are then isolated almost as a final stage in the operation.

Today the technique is usually undertaken with the hepatic pedicle clamped, either totally or on one side. The hepatic pedicle structures are contained within their Glissonian sheaths and when they have been encountered and isolated they are usually divided en masse.

We prefered another approach (1). First, an incision is made at the junction of the hilum with the liver substance of the caudate process i.e. behind the hilum.

This incision is approximately 20 mm in length. A second incision is the made in front of the hilum and parallel to the first incision, extending from the gallbladder bed on the right to the umbilical fissure on the left. The incision is deepened and the liver parenchyma is pushed upwards and away from the hilum in front, in order to expose the Glissonian sheath of the confluence of the hepatic pedicle structures. This dissection in front of the hilum corresponds to the procedure that Couinaud described as "taking down the hilar plate". An index finger is now passed into the incision behind the hilum and the undersurface of the sheath is kept above the finger, which is insinuated between the sheath anteriorly and the caudate process posteriorly until the superior part of the previously dissected sheath is reached. A large curved clamp is then used to pass a tape around this region of the confluence. Traction on the tape tends to exteriorize both the right and left main sheaths. Tapes can now be passed around whichever sheath is required for further dissection.

Once again a large curved clamp is used to place tapes around the various sheaths, which are then dissected. The surgeon's thumb and index finger are at all times useful instruments for dissecting out the sheaths, which are composed of tough fibrous tissue. The ultrasound dissector is also useful for cleaning liver parenchyma off the sheaths. The identity of the sheaths dissected may be known from a knowledge of the intrahepatic anatomy, but variations are so common that confirmation should always be sought by clamping each sheath with a vascular clamp, with the main hepatic pedicle unclamped. Colour changes in the liver substance then identify the region of the liver which the sheath subserves. The sheath to the right medial sector can usually be exposed through this approach, but further dissection to obtain its branches into sheaths for segments V and VIII if often too deep and has to await opening up of the liver parenchyma, usually through the main fissure of the liver. If further access is wanted to the right, then a ventral incision in the posterior aspect of the gallbladder bed, with division of the fibrous tissue in the area (the vesicular plate of Couinaud), can be undertaken.

Even if the sheath to segment VII is not liberated by this approach, it is usually possible to delineate its boundaries by selective clamping of the sheath to the right medial sector (segment V and VIII) and the sheath or sheaths to segment VI.

Provided the technique is carried out with the hepatic pedicle clamped bleeding is not a problem, except occasionally from the incision in the caudate process, behind the hilum. This is hepatic venous bleeding from the caudate lobe and it usually stops spontaneously.

Gotoh *et al.* work present a limited approach to those glissonian sheaths. They should use a larger approach which permits a selective delineation of sectors and segments.

### REFERENCE

1. Launois B, Jamieson G.G. (1992) The posterior intrahepatic approach for hepatectomy or removal of segments of the liver. *Surg. Gynecol. Obstet.*, **174**, 155–158.

Prof. B Launois
Centre Hospitalier & Universitaire de Rennes
Rue Henri le Guilloux
35033 Rennes Cedex
France

